# Supplementary figures and images for: Tracking N- and C-termini of C. elegans polycystin-1 reveals their distinct targeting requirements and functions in cilia and extracellular vesicles
Source: PLoS Genet. 2022 Dec 27;18(12):e1010560. doi: 10.1371/journal.pgen.1010560 (PMC9829181; doi:10.1371/journal.pgen.1010560)

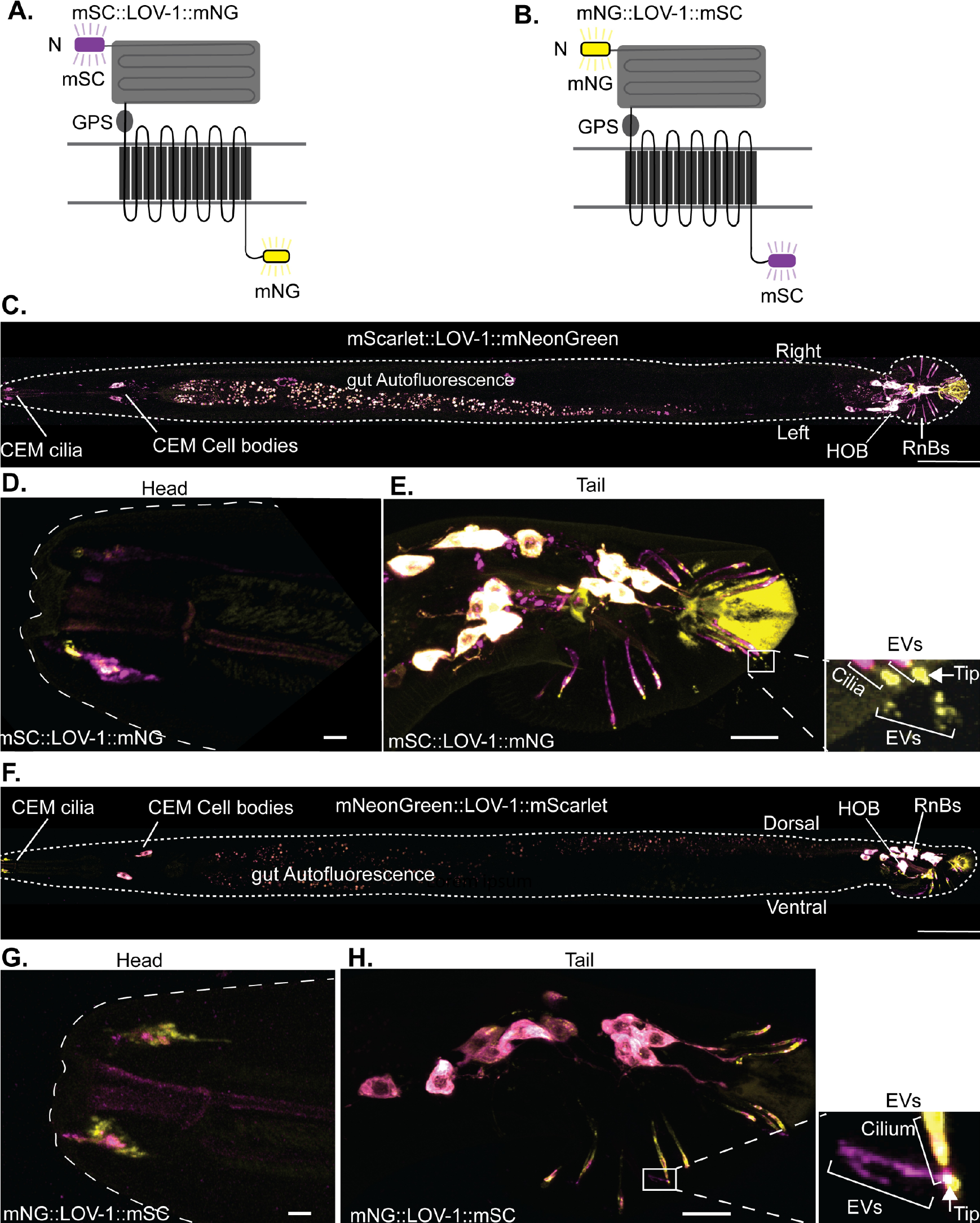

Supplement: S1 Fig — A) Diagram of mSC::LOV-1::mNG. B) Diagram of mNG::LOV-1::mSC. C) 40X tile scan of a whole C. elegans male expressing mSC::LOV-1::mNG. Scale bar is 50 μm. D) Z-projection of male head of worm expressing mSC::LOV-1::mNG. E) Z-projection of male tail of worm expressing mSC::LOV-1::mNG. Inset shows LOV-1::mNG EVs released from the cilia tips of RnB neuronal cilia. F) 40X tile scan of a whole male worm expressing mNG::LOV-1:: mSC. Scale bar is 50 μm. E) Z-projection of male head of worm expressing mNG::LOV-1::mSC. G) Z-projection of male tail of worm expressing mNG::LOV-1::mSC. Inset shows LOV-1::mSC EVs released from the cilia tips of RnB neuronal cilia. The signal in the green channel at the ciliary tip is autofluorescence of the cuticular pore. If we reduce intensity, auto-fluorescent signal is absent but EVs cannot be imaged. D, E, G, H) Scale bars for head and tail images are 2 μm and 10 μm respectively. (TIF) [file pgen.1010560.s001.tif]

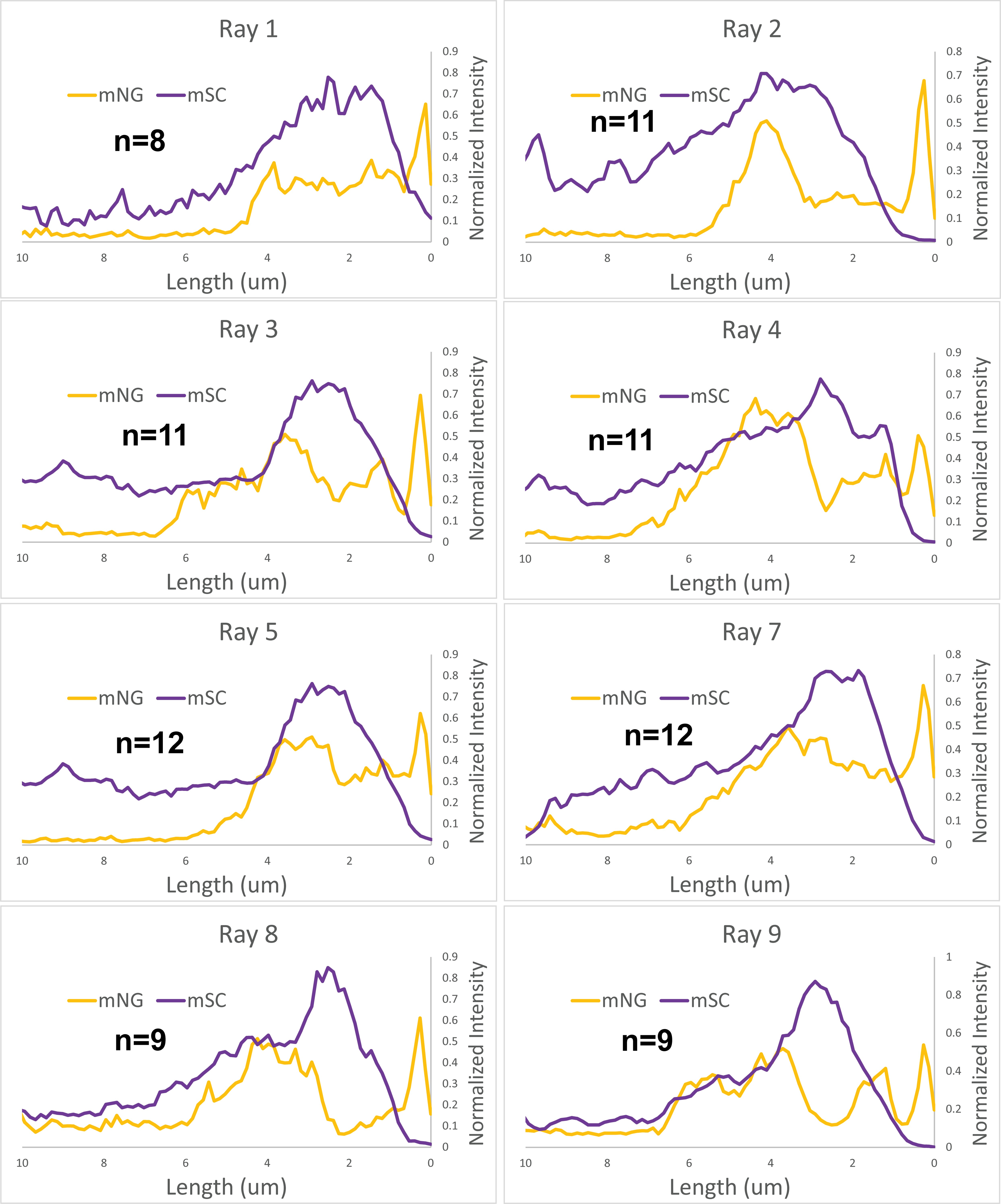

Supplement: S2 Fig — NTM LOV-1 (mSC::LOV-1) is enriched at the distal dendrite, cilia base, and cilia proper but is absent from cilia tips. CTM LOV-1 (LOV-1::mNG) is enriched at the cilia base and cilia tip but not the distal dendrite. n = number of ray neurons measured. (TIF) [file pgen.1010560.s002.tif]

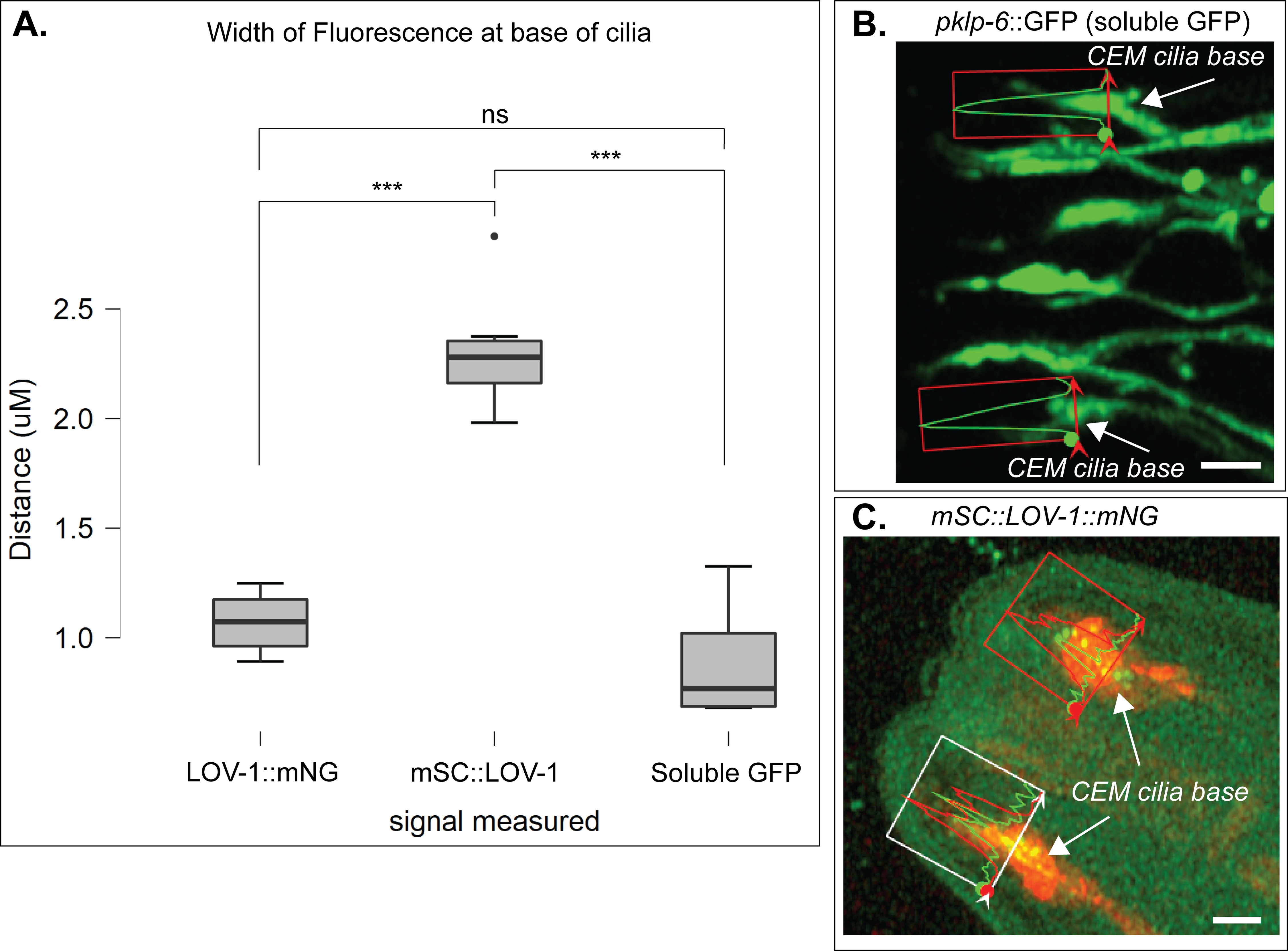

Supplement: S3 Fig — A) Comparison of the width of fluorescent signal at the base of cilia in mSC::LOV-1::mNG worms compared to a soluble GFP ciliated neuronal marker. Statistics performed was an ANOVA with Tukey post-hoc analysis for multiple comparisons. B) Representative image of pklp-6::GFP in the male head. CEM and IL2 cilia are visible. Width measurements were limited to the CEM cilia base region. C) Representative image of mSC::LOV-1::mNG used for signal width measurements. LOV-1::mNG and CEM-expressed soluble GFP correlate in width, thus mSC::LOV-1 must be released outside the CEM cilium. N = 6, 6, and 5 for LOV-1::mSC, mSC::LOV-1, and soluble GFP respectively. Statistics performed using one-way ANOVA and Tukey’s post hoc test. Scale bars are 2 μm. (TIF) [file pgen.1010560.s003.tif]

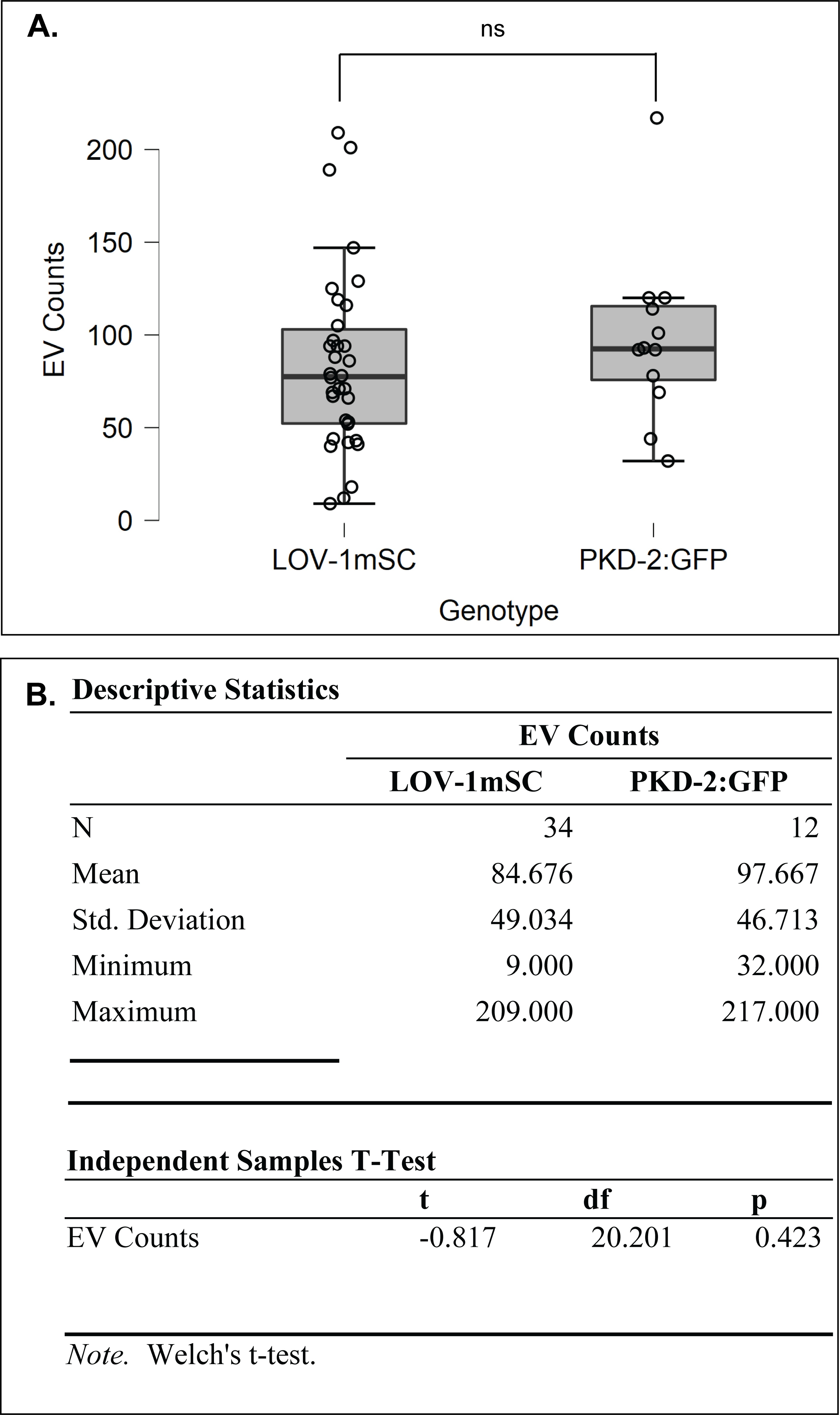

Supplement: S4 Fig — Slightly different EV counts are due to background and/or EV movement during imaging. EV counts were done using an automated counting plugin, ComDet, in ImageJ. Stats generated in JASP and Welch’s T-test was performed. (TIF) [file pgen.1010560.s004.tif]

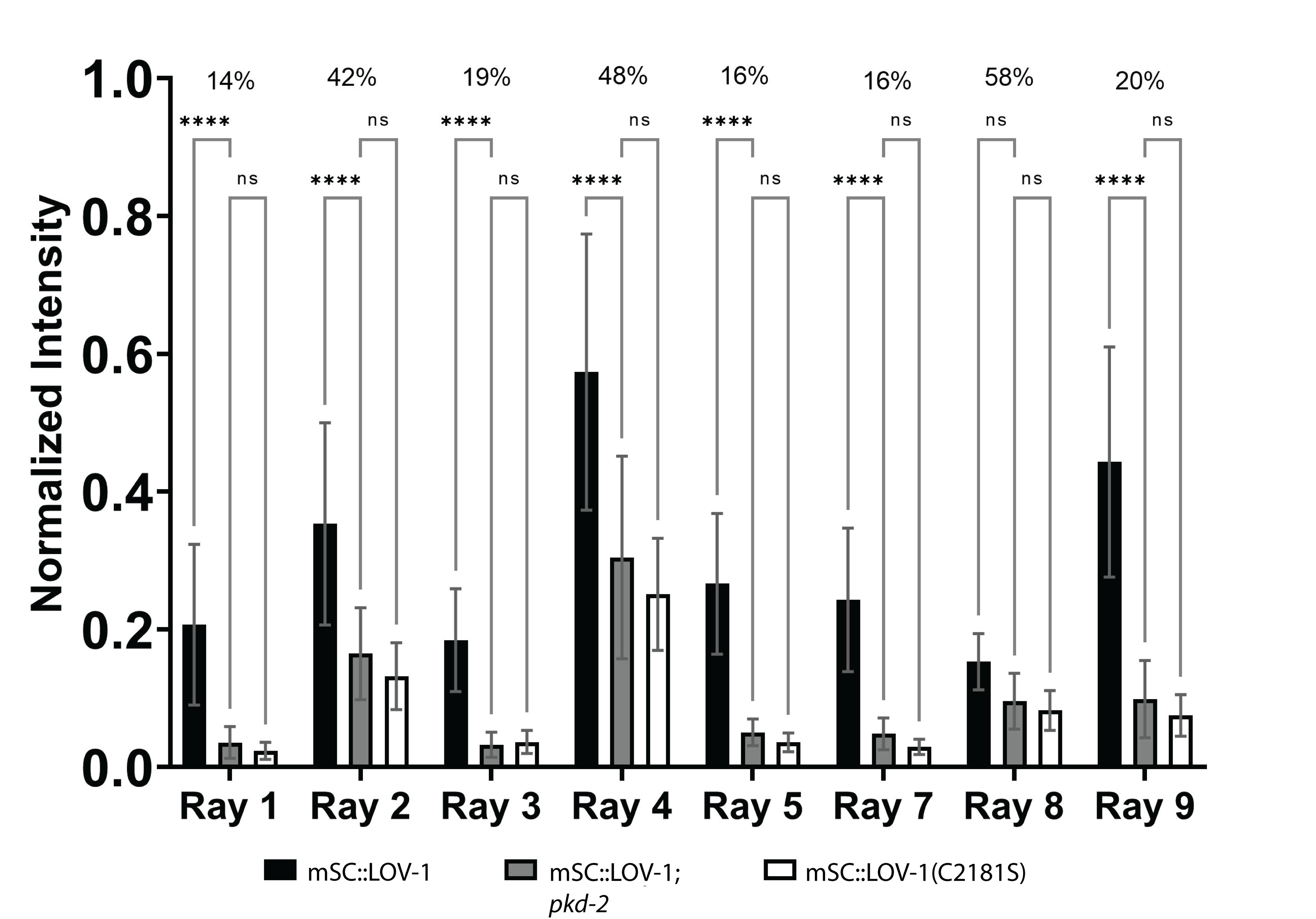

Supplement: S5 Fig — Fluorescence profiling of NTM LOV-1 (mScarlet) in ray cilia of worms expressing mSC::LOV-1::mNG in WT, pkd-2(sy606), or msc::lov-1(c2181s)::mng backgrounds. Percentages above the graph represent the percent reduction of signal in the mutant strains compared to WT. Rays 2, 4, and 8 (the ventral rays) show an enrichment in the mutant strains when compared to the other rays. Statistics performed using 2-way ANOVA and Tukey’s multiple comparisons tests. Sample sizes provided in source data. (TIF) [file pgen.1010560.s005.tif]

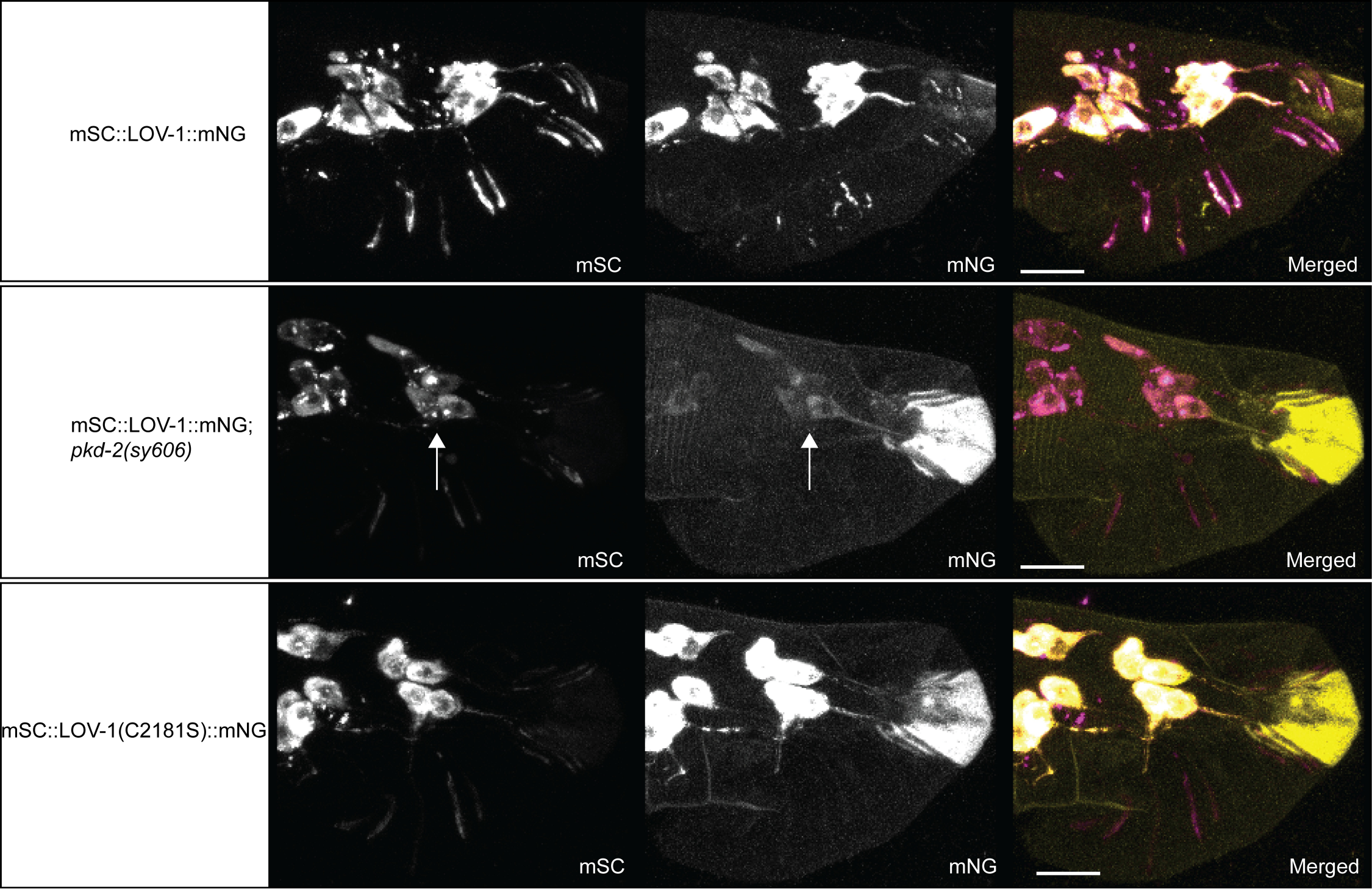

Supplement: S6 Fig — (TIF) [file pgen.1010560.s006.tif]

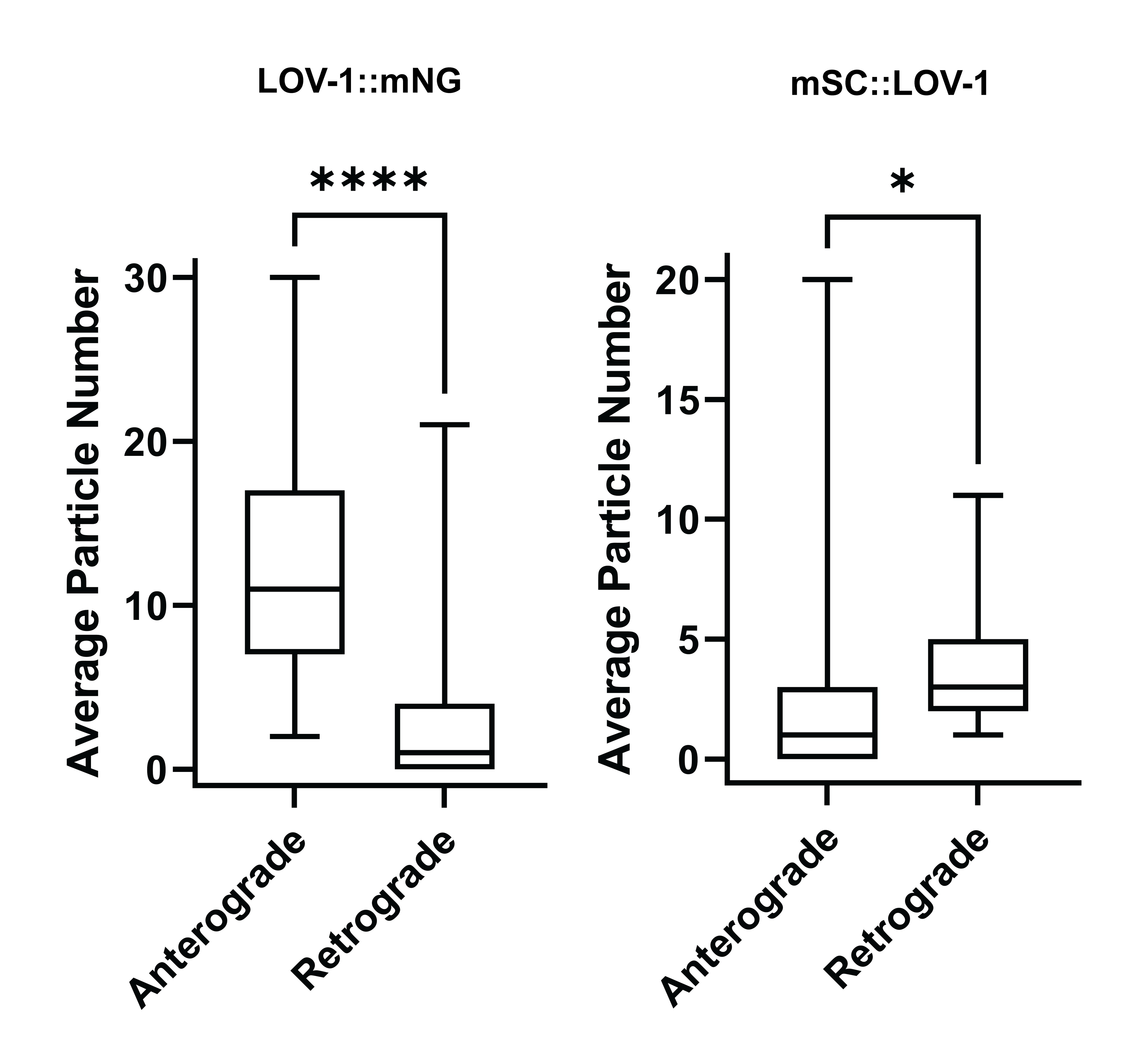

Supplement: S7 Fig — CTM LOV-1::mNG transport occurs more frequently in the anterograde direction, whereas NTM mSC::LOV-1 transport occurs more frequently in the retrograde direction in dendrites. N = 46 and 56 for CTM LOV-1::mNG and NTM mSC::LOV-1 respectively. Statistics performed was unpaired t-test. (TIF) [file pgen.1010560.s007.tif]

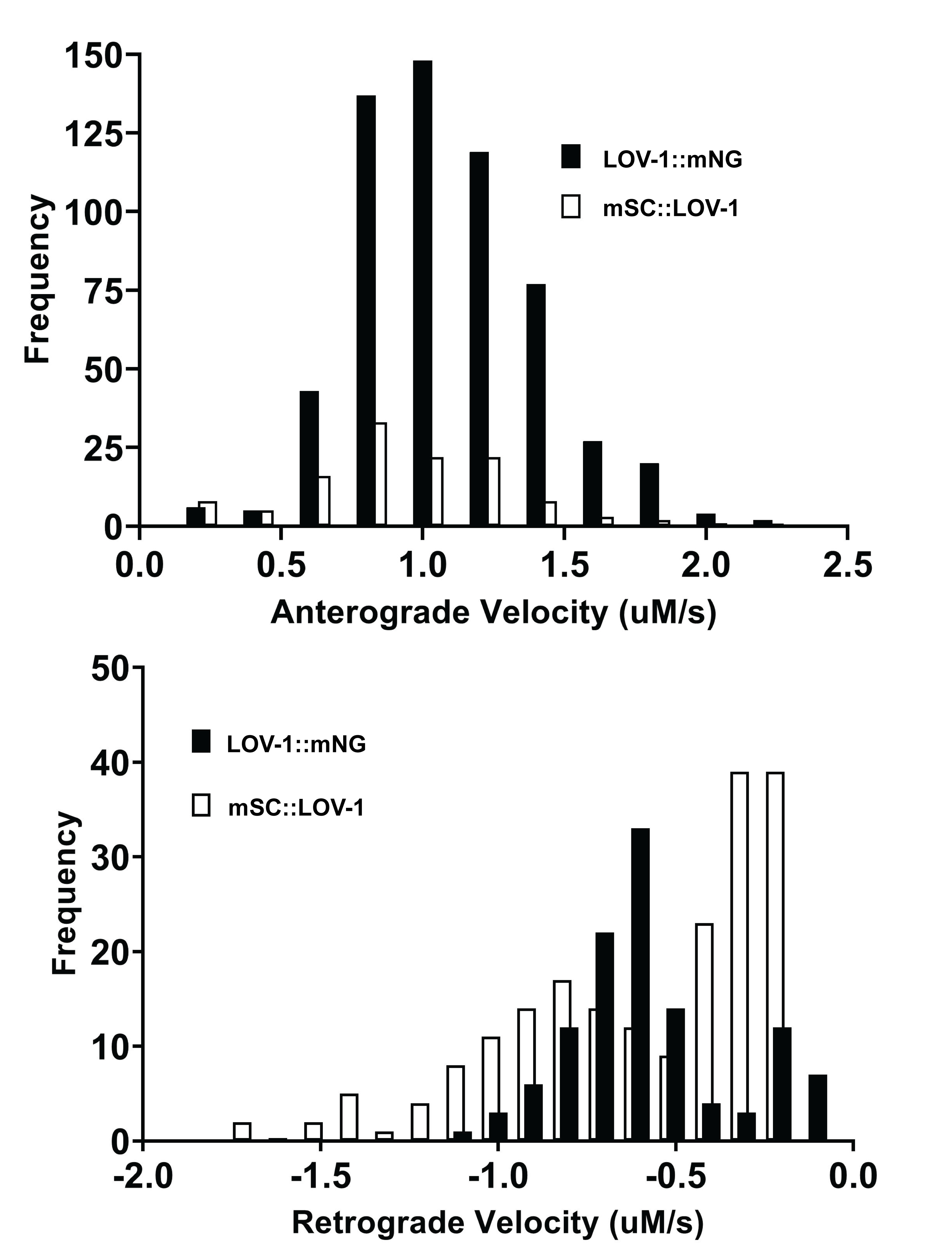

Supplement: S8 Fig — (TIF) [file pgen.1010560.s008.tif]

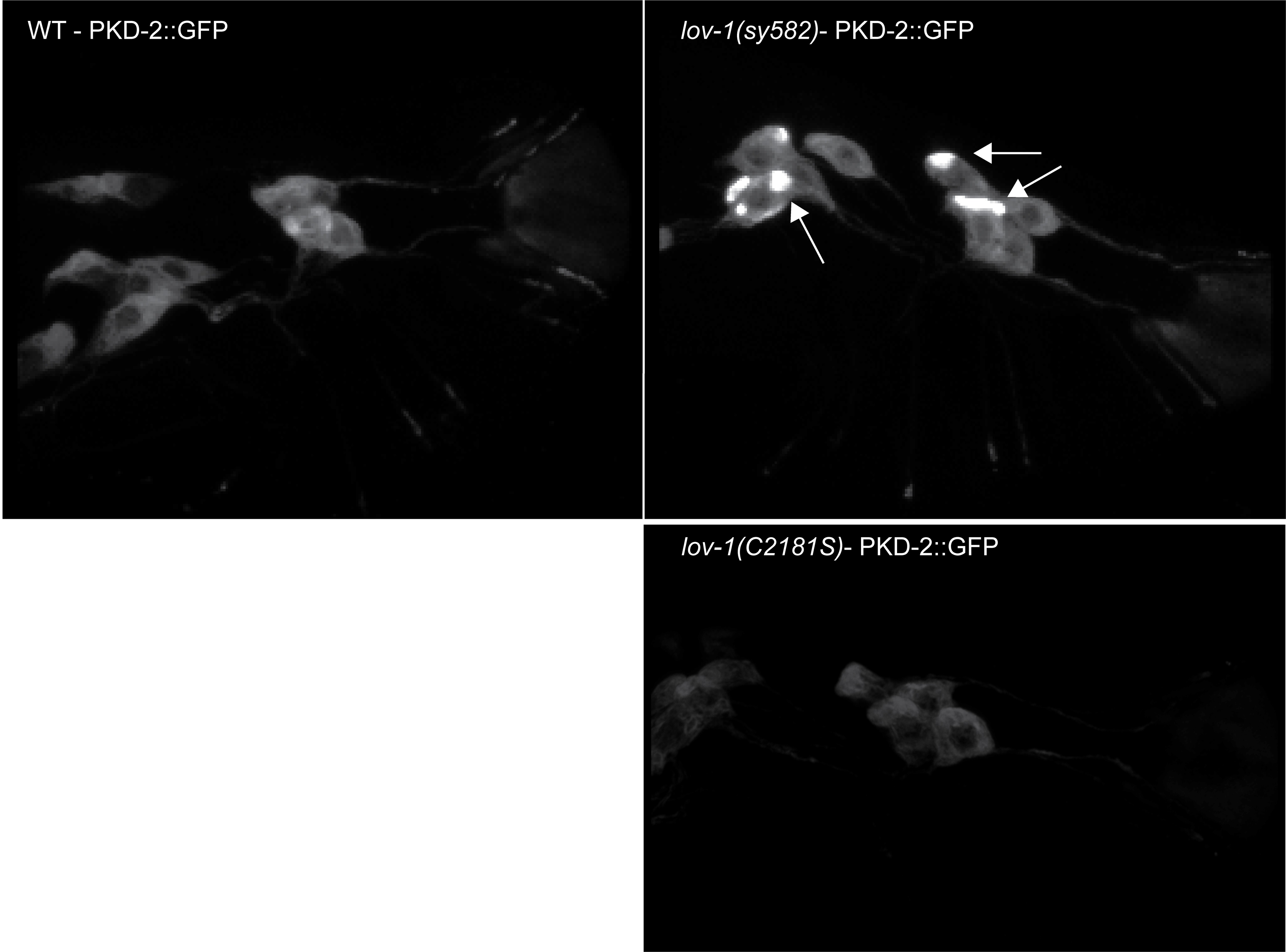

Supplement: S9 Fig — (TIF) [file pgen.1010560.s009.tif]

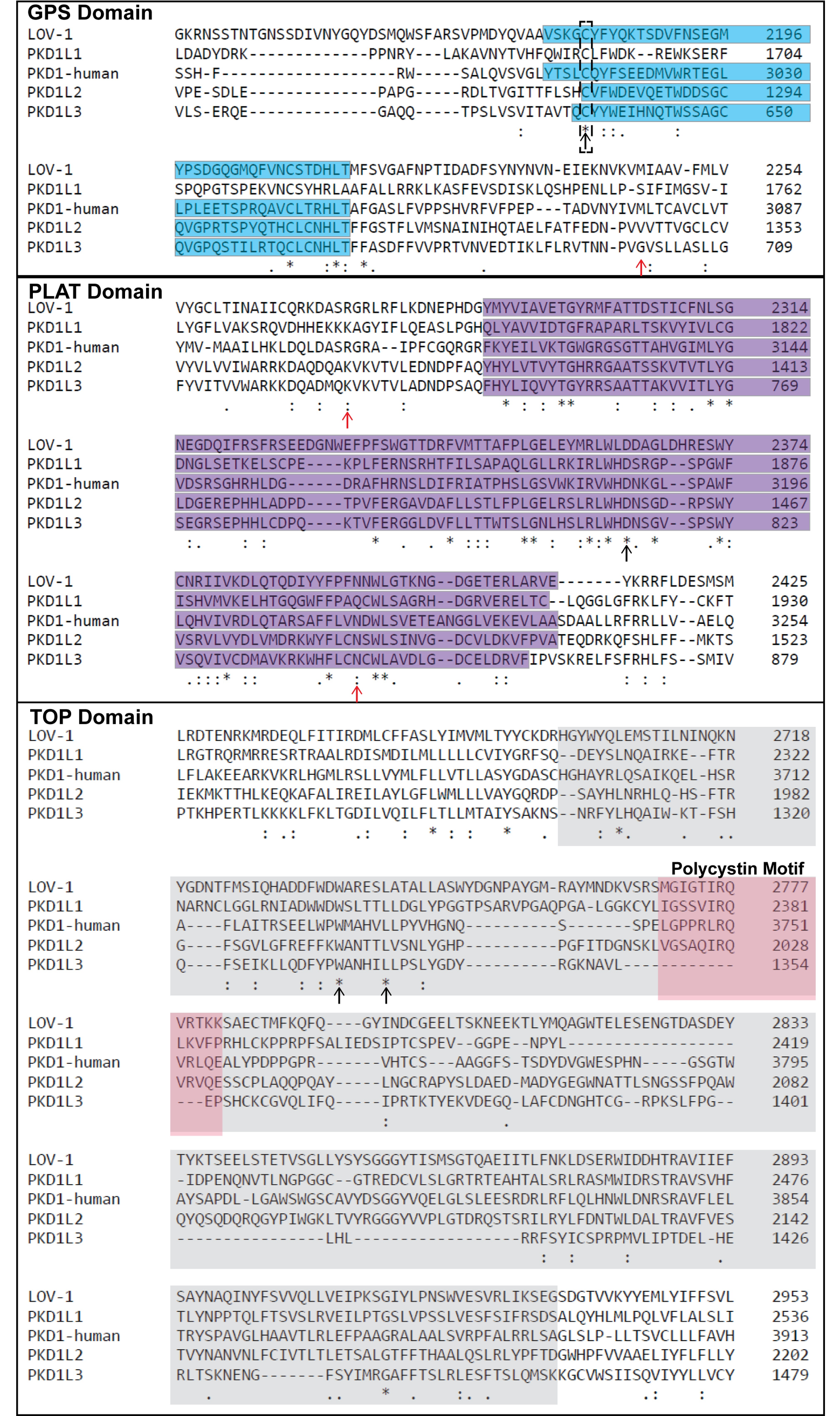

Supplement: S10 Fig — GPS domain highlighted in blue, PLAT domain in magenta, TOP domain in grey, and the polycystin motif in red. Black arrows indicate conserved residues among all aligned proteins that are associated with ADPKD missense mutations in humans. Red arrows indicate residues that are only conserved among PKD1 and LOV- that are associated with ADPKD missense mutations in humans. The dashed box indicates the conserved Cysteine residue of the GPS that was mutated in this study (C2181S). (TIF) [file pgen.1010560.s010.tif]

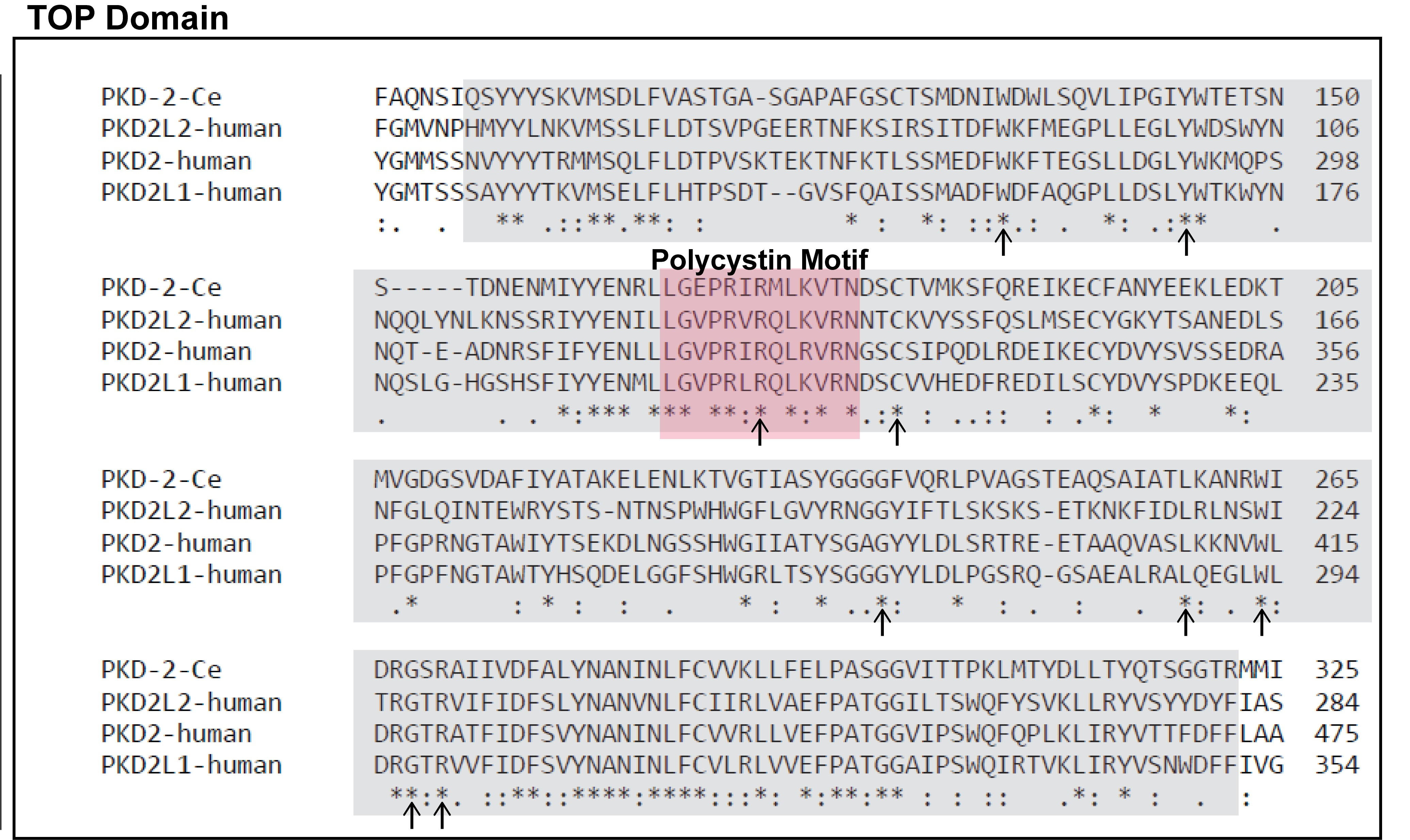

Supplement: S11 Fig — TOP domain in grey and the polycystin motif in red. Black arrows indicate conserved residues among all aligned proteins that are associated with ADPKD missense mutations in humans. (TIF) [file pgen.1010560.s011.tif]

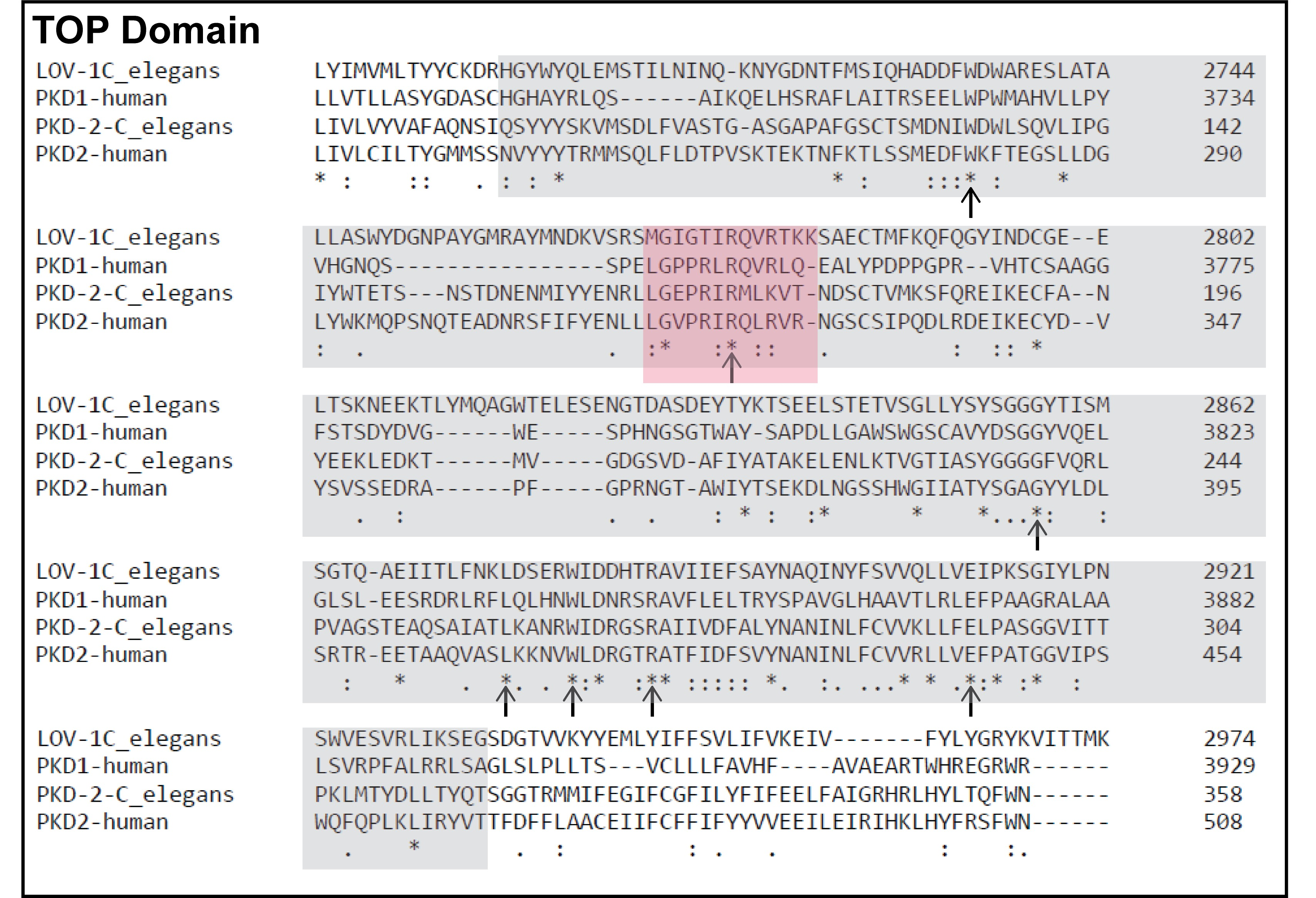

Supplement: S12 Fig — The polycystin motif is highlighted in red. Black arrows indicate conserved residues among all aligned protein domains that are associated with ADPKD missense mutations in humans. These residues may be mutated in human PC1, PC2, or both. (TIF) [file pgen.1010560.s012.tif]

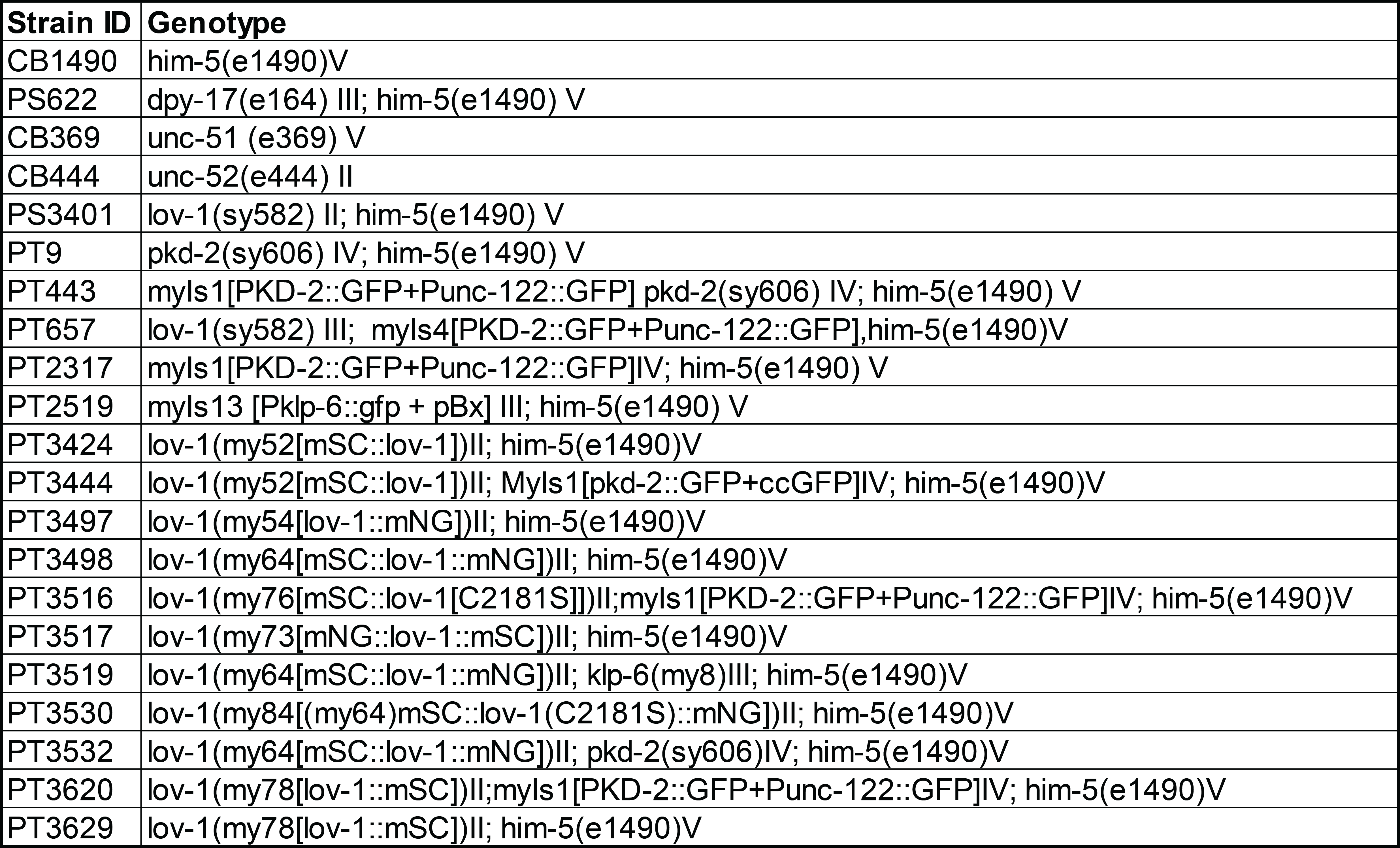

Supplement: S1 Table — All newly generated strains are available upon request. (TIF) [file pgen.1010560.s013.tif]
