# Supplementary material for: Tracking N- and C-termini of C. elegans polycystin-1 reveals their distinct targeting requirements and functions in cilia and extracellular vesicles
Source: PLoS Genet. 2022 Dec 27;18(12):e1010560. doi: 10.1371/journal.pgen.1010560 (PMC9829181; doi:10.1371/journal.pgen.1010560)
Supplement: S2 Table — (DOCX) [file pgen.1010560.s014.docx]

Table 2: DNA and RNA sequences used

| **crRNA homology sequence** |  | color code |
| --- | --- | --- |
| *lov-1 CTM* | GAAGTGGCGGCTAAACGATG **TGG** (Forward Strand) | **homology** |
| *lov-1 NTM* | **CCA** ACTTCTTGACGGGATCGCAA (Reverse strand) | **PAM** |
| *lov-1(C2181S)* | CAAGTCGCTGCAGTGAGTAA **AGG** (Forward Strand) | Silent mutation |
|  |  | Flexible Linker |
| **pdsDNA Primers** |  | **mneongreen** |
| *lov-1::mneongreen* |  |  |
| Long_Forward | CTGGACCAAAGAGATTCCAGAAGTGGCGGCTAAACGAC GTCGAGAAAGATGGAGGTGGCGGATCTGGAGGTGGAGG CTCTGGAGGAGGTGGATCTATGGTGTCGAAGGGAGAAG A |  |
| Long_Reverse | GAATGGATGAACTCTACAAGTAGTTTTTGAGGGCTACCTA  ATTTGGATATCAAGAATA |  |
| Short_Forward | ATGGTGTCGAAGGGAGAAGA |  |
| Short_Reverse | GAATGGATGAACTCTACAAGTAG |  |
| *mscarlet-I::lov-1* |  |  |
| Long_Forward | GAAGGAAAAGCTGAATAAAACTACTTTTAAGATACAAATT  GATGGAATGGTCAGCAAGGGAGAGG |  |
| Long_Reverse | GGAATGGACGAGCTCTACAAGGGAGGTGGCGGATCTGG AGGTGGAGGCTCTGGAGGAGGTGGATCTTTGCATTAtCA gCTTCTTGACGGGATCGCAACTTTTCGATTAGACAACG |  |
| Short_Forward | AAATTGATGGAATGGTCAGC |  |
| Short_Reverse | GGATCTTTGCATTATCAGCT |  |
| *lov-1::mscarlet-I* |  |  |
| Long_Forward | CTGGACCAAAGAGATTCCAGAAGTGGCGGCTAAACGAcG TcGAGAAAGATGGAGGTGGCGGATCTGGAGGTGGAGGC TCTGGAGGAGGTGGATCTATGGTCAGCAAGGGAGAGGC |  |
| Long_Reverse | TATTCTTGATATCCAAATTAGGTAGCCCTCAAAAACTACTT  GTAGAGCTCGTCCA |  |
| Short_Forward | ACGTCGAGAAAGATGGAGGT |  |
| Short_Reverse | CTACTTGTAGAGCTCGTCCA |  |
| *mneongreen::lov-1* |  |  |
| Long_Forward | GAAGGAAAAGCTGAATAAAACTACTTTTAAGATACAAATT  GATGGAATGGTGTCGAAGGGAGAAGA |  |
| Long_Reverse | CGTTGTCTAATCGAAAAGTTGCGATCCCGTCAAGAAGcT GaTAATGCAAAGATCCACCTCCTCCAGAGCCTCCACCTC CAGATCCGCCACCTCCCTTGTAGAGTTCATCCATTC |  |
| Short_Forward | AATTGATGGAATGGTGTCGA |  |
| Short_Reverse | AGCTGATAATGCAAAGATCC |  |
|  |  |  |
| **ssODNs** |  |  |
| *lov-1(C2181S)* | **GCACGAAGTGTACCAATGGATTATCAAGTCGCTGC**CGTC TGGAAAGGA**TCC**TAC **TACTTCTATCAGAAAACATCGGATGTCTTCAATTCTG**  **(TGT --> TCC = Cysteine --> Serine)**  BamHI site |  |
|  |  |  |
| **Genotyping Primers** |  |  |
| *lov-1::mneongreen* |  |  |

| Outside | Forward:CAGACAAAACGTCGCTTGGG  Reverse:TGACCCATCATGCCTTTGTTC |  |
| --- | --- | --- |
| Inside | Forward:CAGACAAAACGTCGCTTGGG  Reverse:TGTGCGATCCCTCGTATGTG |  |
| *mscarlet-I::lov-1* |  |  |
| Outside | Forward:CCGCCTTTTCGCTTTTCGAC  Reverse:CCGCCTTTTCGCTTTTCGAC |  |
| Inside | Forward:CCGCCTTTTCGCTTTTCGAC  Reverse:AGTAGTCTGGGATGTCGGCT |  |
| *lov-1::mscarlet-I* |  |  |
| Outside | Forward:CAGACAAAACGTCGCTTGGG  Reverse:TGACCCATCATGCCTTTGTTC |  |
| Inside | Forward:CAGACAAAACGTCGCTTGGG  Reverse:AGTAGTCTGGGATGTCGGCT |  |
| *mneongreen::lov-1* |  |  |
| Outside | Forward:CCGCCTTTTCGCTTTTCGAC  Reverse:CCGCCTTTTCGCTTTTCGAC |  |
| Inside | Forward:CCGCCTTTTCGCTTTTCGAC  Reverse:TGTGCGATCCCTCGTATGTG |  |
| *lov-1(C2181S)* | Forward: TATGTGAACGGCAGGGGAAG  Reverse:GTGGTTCCCCATGAAAACGG Restriction Digest: BamHI |  |
|  |  |  |
| **Gene Fusion Sequences** |  |  |
| *lov-1::mneongreen* | **CTGGACCAAAGAGATTCCAGAAGTGGCGGCTAAACG**AC  G**TCG**AGAAAGATGGAGGTGGCGGATCTGGAGGTGGAGG CTCTGGAGGAGGTGGATCT**ATGGTGTCGAAGGGAGAAG AGGATAACATGG** |  |
| *mscarlet-I::lov-1* | **ACGTCACTCCACCGGAGGAATGGACGAGCTCTACAAG**G  GAGGTGGCGGATCTGGAGGTGGAGGCTCTGGAGGAGG TGGATCTTTGCATTA**TCA**G**CTTCTTGACGGGATCGCAAC TTTTCGATTAGACAACG** |  |
| *lov-1::mscarlet-I* | **CTGGACCAAAGAGATTCCAGAAGTGGCGGCTAAACG**AC  G**TCG**AGAAAGATGGAGGTGGCGGATCTGGAGGTGGAGG CTCTGGAGGAGGTGGATCT**ATGGTCAGCAAGGGAGAGG CAGTTATCAAGGAGTTCA** |  |
| *mneongreen::lov-1* | **CAGATGTGATGGGAATGGATGAACTCTACAAG**GGAGGT  GGCGGATCTGGAGGTGGAGGCTCTGGAGGAGGTGGAT CTTTGCATTA**TCA**G**CTTCTTGACGGGATCGCAACTTTTCG ATTAGACAACG** |  |
