# Supplementary material for: Tracking N- and C-termini of C. elegans polycystin-1 reveals their distinct targeting requirements and functions in cilia and extracellular vesicles
Source: PLoS Genet. 2022 Dec 27;18(12):e1010560. doi: 10.1371/journal.pgen.1010560 (PMC9829181; doi:10.1371/journal.pgen.1010560)
Supplement: Attachment — Submitted filename: Reviewer comments rebuttal11172022.pdf [file pgen.1010560.s020.pdf]

Reviewer #1:

We thank Reviewer 1 for stating that our manuscript is a "tour de force" and their mammalian PKD biologist's point-of-view, which we now include as a new discussion point:

The observation that the N-terminus of LOV-1 is stable and can be appropriately targeted in the context of mutations in the GPS domain and the absence of PKD-2 is important from a mammalian PKD biologist's point-of-view. These findings suggest that mutations in the C-terminus of the PKD1 gene or in PKD2 gene may give rise to an N-terminal ectodomain. This pathological N-terminal ectodomain may have the ability to bind ligands but lack signaling activity – a dominant negative scenario. This may be very important for our understanding ADPKD: humans (but not mice) naturally make an N-terminal truncation product that might be ligand binding -- Trunc_PC1 • DOI: 10.1681/ASN.2018040442

We also corrected typos.

Reviewer #2:

1. The experiments suggest that the N-terminus of LOV-1 may play a PKD-2 independent role in inhibiting the ascr#3 response. Is overexpression of just the N-terminus of LOV-1 in a lov-1; pkd-2 double mutant sufficient to decrease their ascr#3 response?
LOV-1 is a 3284 amino acid protein, with the N-terminus being 2214 amino acids. Before the advent of CRISPR, the large size of the *lov-1* locus prohibited structure-function-localization analysis and the experiment Reviewer 2 suggests is still technically challenging and beyond the scope of this manuscript. We previously showed that an extrachromosomal array expressing a GFP-tagged LOV-1 fragment containing the first 991 amino acids dominantly interfered with wild-type response and vulva location behaviors and did not rescue *lov-1* null response and location of vulva defects (Barr and Sternberg 1999), which is consist with an important function of the N-terminus. To Reviewer 2s suggestion, we now include a Future Direction paragraph addressing the function of the LOV-1 N-terminus using CRISPR to generate NTM only and CTM only strains.

2. Could the authors speculate a bit on the interesting ECM localization of the LOV-1 N-terminal domain?
We have two speculations, but we are only comfortable adding point 1 to the discussion of this manuscript, as we have supporting data in a bioRxiv preprint. We look to Reviewer 2 and the Editor for guidance here.

1) We have evidence for extracellular matrix (ECM) playing a role in *C. elegans* polycystin signaling and EV release. EVs are components of the ECM and EVs themselves may carry ECM proteins and ECM-modifying enzymes[1,2]. These ECM-loaded EVs may serve as essential modifiers of the existing ECM composition, and thus support signaling between ECM and the surrounding cells. *Chlamydomonas* ciliary EVs carry ECM proteins and ECM-degrading proteases [3,4]. ECM is important for neuronal anatomy and organization of the brain and nervous system [5]. Abnormal ECM and fibrosis are observed in ciliopathies and neurodegenerative diseases including Alzheimer's disease. We found that the *mec-1*, *mec-5*, and *mec-9* genes encoding ECM components regulate

PKD-2 and LOV-1 ciliary localization, ciliary EV shedding and neuron-glia interactions[6]. MEC-1 and MEC-9 contain EGF/Kunitz domains and MEC-5 is a unique collagen [7]. One interesting possibility is that the LOV-1 N-terminus interacts with these ECM components to regulate sensory organ homeostasis.

1 Rilla, K. *et al.* Extracellular vesicles are integral and functional components of the extracellular matrix. *Matrix Biol* (2017).
2 Rackov, G. *et al.* Vesicle-Mediated Control of Cell Function: The Role of Extracellular Matrix and Microenvironment. *Front Physiol* **9**, 651 (2018). PMC5996101.
3 Wood, C. R., Huang, K., Diener, D. R. & Rosenbaum, J. L. The cilium secretes bioactive ectosomes. *Curr Biol* **23**, 906-911 (2013).
4 Long, H. *et al.* Comparative Analysis of Ciliary Membranes and Ectosomes. *Curr Biol* **26**, 3327-3335 (2016). PMC5173405.
5 Gardiner, N. J. Integrins and the extracellular matrix: key mediators of development and regeneration of the sensory nervous system. *Dev Neurobiol* **71**, 1054-1072 (2011).
6 De Vore, D. M., Knobel, K. M., Nguyen, K. C. Q., Hall, D. H. & Barr, M. M. Extracellular matrix regulates morphogenesis and function of ciliated sensory organs in Caenorhabditis elegans. *bioRxiv* (2018).
7 Emtage, L., Gu, G., Hartwieg, E. & Chalfie, M. Extracellular proteins organize the mechanosensory channel complex in C. elegans touch receptor neurons. *Neuron* **44**, 795-807 (2004).

2) The LOV-1 N-terminus may have an attenuating effect on ascaroside detection. This could be due to its ECM localization and its adhesive mucin-like domains. We speculate that the LOV-1 N-terminal domain may inhibit signaling between the CEM cilium, the CEP cilium, and/or glial cells.

3. Narayan et al (PNAS 2016) previously showed that the 4 CEM neurons show different responses to ascarosides that are not correlated with their anatomical positions/identities. While synaptic modulation has been suggested to play a role in this, an interesting possibility might be that LOV-1 is differentially processed and/or localized to individual CEMs to alter their response profiles. Having an endogenous reporter-tagged allele of LOV-1 should allow the authors to examine this possibility and would be a strong addition to the paper.

This is a great suggestion. We have some interesting preliminary findings on the stochastic localization of LOV-1 in CEM neuronal cilia. We revisited images of males used for a different experiment and observed differences in the localization of LOV-1 in different CEM neurons. Unfortunately, our original datasets did not capture/align animals and do not allow us to distinguish cilia of CEMDR, CEMDL, CEMVR, and CEMVL neurons. We included this as a future direction in a conclusion paragraph. We also include some very preliminary data here for Reviewer 2.

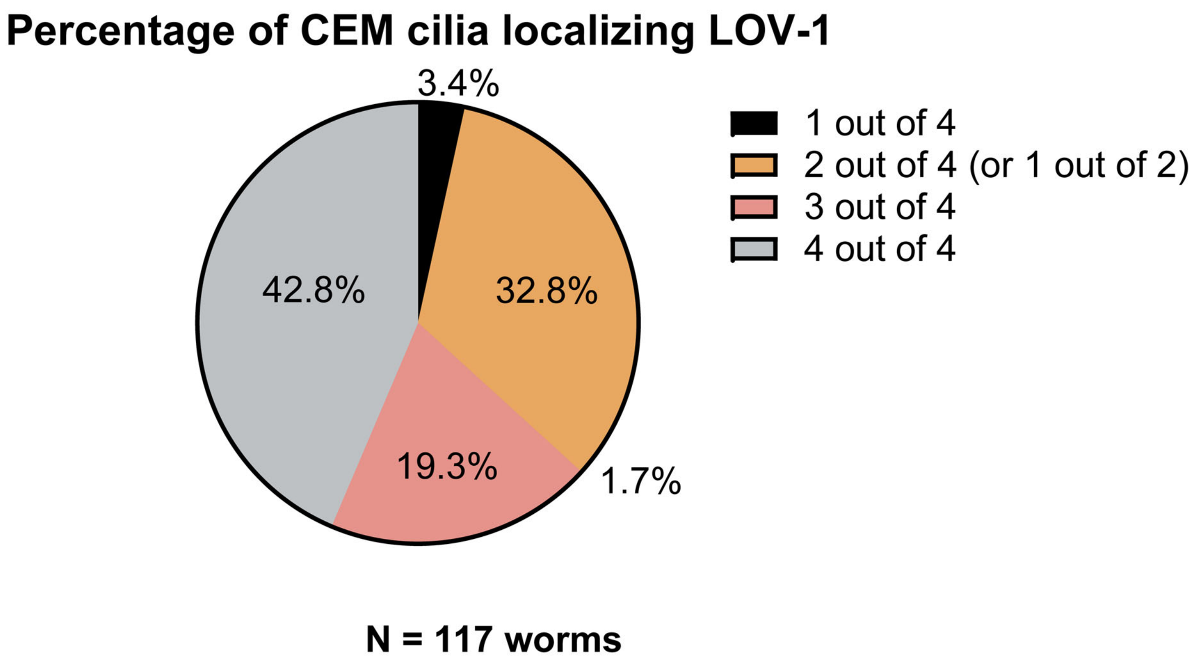

Percentage of CEM cilia localizing LOV-1. Data was collected from all images taken containing the head region of the adult *C. elegans* male in LOV-1 CRISPR strains that were otherwise WT. Males were not oriented in any specific way and cell bodies were not captured, therefore orientation cannot be determined to identify specific neuronal cell bodies (dorsal, ventral, left, right). Counts of CEM neurons with visible ciliary enrichment of LOV-1 were scored per total CEM neurons captured in each image. In some instances, only one side of the animal was scorable (1 out of 2).

3.4% of all males analyzed contained only one CEM cilia that localized LOV-1. 32.8% of all males analyzed contained 2 out of 4 or 1 out of 2 CEM cilia that localized LOV-1. 19.3% of all males analyzed contained three CEM cilia that localized LOV-1. 42.8% of all males analyzed contained LOV-1 in all CEM cilia observed. Data is biased for 50% and 100% of CEMs localizing LOV-1 because we were focusing on LOV-1 localization in cilia and would not image males with 0% of CEM with LOV-1. Future data collection will be unbiased and with animals oriented to identify cell bodies.

4. GPS disruption - Can the authors comment on if they suspect that cleavage is completely blocked or just attenuated in this mutant from their imaging?
We address this question in the results section:
"These data suggest either incomplete cleavage in the C2181S mutant or other cleavage sites are present that are not disrupted by the C2181S substitution. The latter is likely since the LOV-1::mNG signal is completely absent in dendrites and cilia. In support of the possibility, other cleavage sites are observed in human PC1 including an N-terminal site upstream of the GPS in an alternative splice form (Trunc_PC1) [27, 28]."

If not, could Westerns be useful here? It looks like there are some regions that are more enriched for N- vs C-terminus in Figure 3B. Is the mutant protein just unstable, here also westerns might be useful in simultaneously visualizing the cleavage and levels.

We now include a Limitation of Study section with this point: lines 242-251 in tracked version. This fit best in the results section describing these limitations.

To determine how the C2181S mutation affects the LOV-1 protein, we tried to do westerns using two different methods to no avail. First, we did westerns with isolated EVs enriched for PKD-2::GFP using methods described in Nikonorova et al (2022). We could not detect PKD-2::GFP using highly sensitive GFP antibodies and using super-sensitive ECL reagent. We also tried westerns by picking 1,000 adult males, flash freezing in liquid nitrogen, and crushing in lysis buffer. This was insufficient material to see even with very good mNG nanobody to LOV-1::mNG. In the future, we are considering using 100% male producing strain (new strain using auxin inducible degron to select for males only). We would use nanotrap beads to immunoprecipitate wild-type and C2181S LOV-1::mNG from lysates of millions of males.

Minor:
1. Please define NTM and CTM in the abstract - done
2. “is” missing from last sentence in abstract - done
3. Figure 4A: graph labels are too small and are unreadable - done

Reviewer #3:

1) In Figure 2, the authors claim that the scarlet and GFP fluors colocalize to the ER but there is not ER marker shown to confirm that localization.
We previously showed that PKD-2::GFP and ER-marker TRAM-1 colocalize in cell bodies of male-specific ciliated neurons (Figure 1H shown here from Bae et al 2006). As LOV-1 and PKD-2 colocalize in cell bodies, we are confident in our conclusion that LOV-1 localizes to the ER. We now specifically reference this previous work that supports our conclusion.

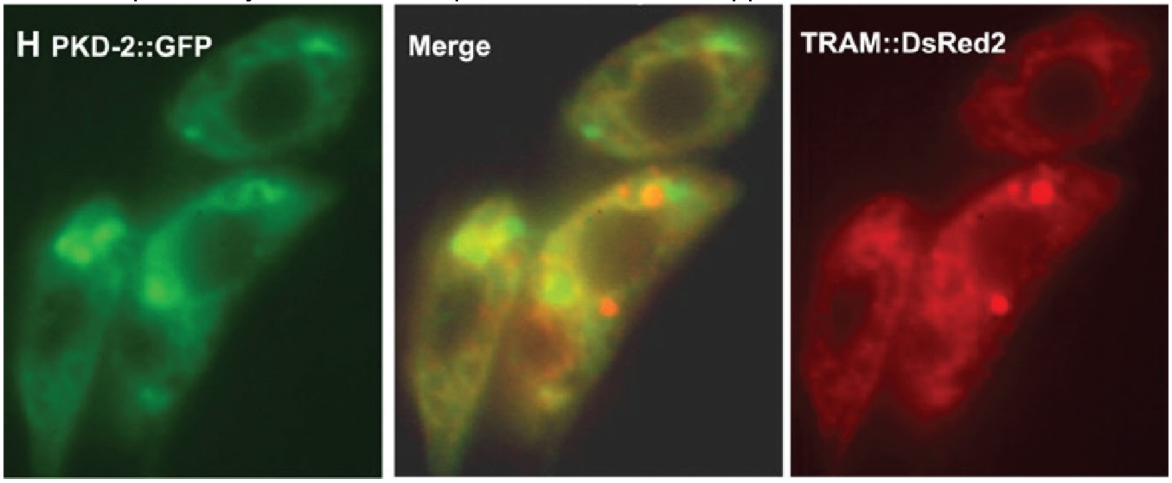

2) Figure 2 and the associated text needs clarification. The figure shows the NTM which is not discussed in the text but is mentioned in the figure legend as being distinct from the CTM- much as Figure 1 just showed. What’s the NTM experiment testing?

This clarification is now addressed in lines 163-168 in tracked version (pasted below).

“Interactions between LOV-1 and PKD-2 were not explored due to the absence of a full-length, functional LOV-1 reporter. To address this knowledge gap, we built strains expressing N-terminally tagged mSC::LOV-1 and C-terminally tagged LOV-1::mSC crossed into an integrated and functional transgenic PKD-2::GFP strain(Fig 2A-B)[24]. N-terminally tagged mSC::LOV-1 demonstrated partial colocalization with PKD-2::GFP. Localization specific to mSC::LOV-1 was observed in the soma, in the dendrites, and surrounding the ciliary base. In addition, only PKD-2::GFP localized to the ciliary tip or cilia-derived EVs. C-terminally tagged LOV-1::mSC and PKD-2::GFP colocalized in the neuronal cell bodies, in dendritic vesicles, at the ciliary base, along the cilium, at the tip, and in environmentally shed ciliary EVs”

Furthermore, while the gap of no full length functional LOV-1 reporter previously being available is clear, it is less clear why the LOV-1::mSC construct would label full length protein (or whether it has) since the GPS is presumably intact enabling cleavage. Is PC1 thought to be incompletely cleaved? What am I missing?

Thank you for bringing this to our attention. We included a more detailed interpretation in the results section lines 232-239 in tracked version (see response to reviewer 2 comment 4).

3) Perhaps for the same reason, I am also confused in the C2181S mutant experiment. Is there complete cleavage? The authors’ state that the mutation is predicted to prevent cleavage so if that is then the prediction is that the tags should co-localize. They don’t so is this disrupting cleavage as predicted? A western probed for each of the tags would make interpretation of these data possible.

See our response to reviewer 2 comment 4.

4) The authors jump around among many distinct statistical tests and it is not clear such a variety is needed: Welch’s, Dunn’s multiple comparison, Dunnett’s T3 multiple comparison, unpaired t-test, one-way ANOVA. Why? Usually Welsh’s is used for data with uneven distribution which this does not appear to be. It is just unclear that so many distinct test are needed especially when the same measurement is being made (eg fig 1 and 5 EV counts). What’s the reasoning behind each test’s selection?

We selected tests because of the differences in data distribution. The data sets were all run through F tests to determine equal or unequal variance. For equal variance, traditional tests were used like ANOVA and student’s T. If the variance was unequal, nonparametric tests were used like Welch’s and Dunnett’s T3. As for comparing Figure 1 to Figure 5, Figure 1 data showed unequal variance because of the large range in the data. In this instance, only 2 sets of data were compared and Welch’s test was sufficient, which is the nonparametric version of the student’s T for sample sizes <50. In Figure 5, there are 3 data sets being compared. Therefore, we used Dunnett’s T3 test to do multiple comparisons for correcting type 1 error in nonparametric (unequal variance) testing. The Dunnett’s test is the post-hoc analysis that uses p-values derived from Welch’s test. To summarize, we are trying to be thorough and conservative in statistical analysis and to eliminate type 1 error. We include all statistical analysis information, including F-tests for equal or unequal variance in supplemental.

Attention should be paid to:

1) The nomenclature the authors use switches around between the text figures and

legends from the actual constructs (mSC::LOV-1::mNG) to shortcuts CTM:LOV-1 (confusing as it doesn't really reflect that the tag is on C-term) to CTM (which tag?). The nomenclature in worms is clear and consistency is always easier to follow. On a related point, I am not sure the NTF/CTF is helpful as it is barely used since the authors only follow the tagged cleaved products. Could just be spelled out when used and avoid more alphabet soup.

Thank you. We corrected this throughout the main text, the figures, and figure legends.

2) Figure 3 (along with legend and associated text) would be easier to follow if the C2181S mutation were used when referring to the mutant and not GPS (which is not a mutation)

Thank you. We also corrected this throughout the main text, the figures, and figure legends.

3) Swapping the tags to test whether either impacts function is a nice control but I think all the labels in SFig1 are incorrect, along with the legend. Additionally, a diagram like Fig 1A showing the swap and parallel figures to 1 C, G and H would make SupFig1 much easier to follow (because the reader would have just seen it in Fig 1).

We are confused by this - all the labels in SFig1 are correct and did not need to be changed. As Reviewer 3 suggested, we added a diagram for each of the tagged version and changed the figure legend.

4) As often happens some of the figures have been modified since the legends were written. A careful, detailed review is needed so that the reader can follow the story. One example in Figure 1 legend is that the adjusted panels on the left are listed under D when they are displayed in panel C. Similarly, the text sometimes does not appear to refer to the correct panel. One example is line 168 in which the EVs are visible in Figure 2B (not referred to in text here).

We missed this and appreciate Reviewer 3's careful reading. We carefully reviewed and corrected discrepancies.

5) In Figure 7 for clarity, the authors should consider either swapping steps 6 and 5' (so EV release on bottom and NTM fragment outside cilia on top) OR putting the whole prime pathway along the bottom and the main (non-prime) pathway along the top. It is just not intuitive that 5' specifically follows 4' and that 5, 6 follow 4 at present. I got there but it took several readings of legend and text.

Thank you for this re-organization suggestion. We changed the location of 4' to be along the top so that 3', 4', and 5' are along the top of the cilium. 1, 2, 3, 4, 5 are along the bottom of the cilium. The only label that is not in-line now is 6, which should be apparent that it is part of the PKD complex after EV release from the ciliary tip.
